# Biomedical Engineering Professional Skills Development: The RADx^SM^ Tech Impact on Graduates and Faculty

**DOI:** 10.1109/OJEMB.2021.3070831

**Published:** 2021-04-28

**Authors:** Andrew J. DiMeo, Chipo J. Afamefuna, Skyler J. Ward, Phil Weilerstein, Elias Caro, Max Germer, Alexander J. Carroll

**Affiliations:** North Caroline State University, Raleigh, NC 27695 USA.; CanvasGT Inc, Raleigh, NC 27601 USA; AMB Consultants North Miami Beach, FL 33179 USA.; RADx APF Charleston, SC 29412 USA.; VentureWell, Hadley, MA 01035 USA.; BiocomX, Dana Point, CA 92629 USA.; VentureWell, Hadley, MA 01035 USA.; Vanderbilt University, Nashville, TN 37235 USA.

**Keywords:** Biomedical, education, engineering, entrepreneurship, professional

## Abstract

There are many benefits of the RADx^SM^ Tech initiative worth exploring beyond that of the current acceleration of diagnostic tests being developed and deployed to the nation. One of those benefits has been the impact on work readiness for recent biomedical engineering (BME) graduates who have been hired by RADx Tech as Assistant Project Facilitators (APFs) and to the students and faculty members on applicant teams. This paper includes a literature review of the current status of BME professional skills development in traditional academic and clinical settings. The organizational structure of RADx Tech teams is described, including how recent BME graduates are integral to the process. Opportunities are discussed on how the RADx Tech structural model can be leveraged to improve professional skills education. It is concluded that the RADx Tech organizational structure and process including APFs may be replicable. Further research is planned to explore its impact.

## I. Introduction

There is a substantive body of literature highlighting an educational gap between Biomedical Engineering (BME) theory and professional skills. BME Programs have been making great efforts to fill this gap, particularly over the past two decades. This has resulted in innovative approaches to teach Senior Design courses, a new breed of Professional Master’s Degrees, and widely available Certificate Programs [1]-[3]. However, this theory/practice gap still exists as evidenced by Rivera, *et al*., in their June 2020 ASEE Virtual Conference paper, “Preparing Early Career Biomedical Undergraduates through Investigations of Stakeholder Needs: A Qualitative Analysis” [4].

Providing training and experiences that meet the current needs of industry is a perennial challenge for BME programs. This challenge is exacerbated when considering many BME faculty have limited prior industry experience to draw from [5]. A recent survey of current BME faculty found a general need for improving students’ preparation for working in industry and identified several key areas for improvement including design for manufacturing, verification and validation, and establishment of industry contacts. In that same study, a survey of industry respondents identified the top five skills for new BME hires as problem-solving, interpersonal communication, design experience, team projects, and writing/delivering technical presentations [6]. The modern BME curriculum is designed to address these practical skills. However, many instructors struggle to transmit the nuances of design controls, regulatory pathway to approval, medical economics, reimbursement, and funding strategy without having prior hands-on experience.

One of the unanticipated outcomes of the COVID-19 pandemic was the emergence of a unique opportunity to address this gap in key experiences. In March of 2020, a grassroots collaboration among biomedical faculty, students, and industry emerged in response to the surging COVID-19 pandemic focused initially on ventilator designs and masks for the dwindling hospital supply [7], [8]. Meanwhile, government officials increasingly identified the need to provide significant funding to biomedical innovators. On May 7, 2020, National Institutes of Health (NIH) Director Francis Collins, MD, PhD, testified at a full senate hearing titled, “Shark Tank: New Tests for COVID-19” [9]. At this hearing, the NIH initiative for Rapid Acceleration of Diagnostics (RADx^SM^) was announced. This effort, specifically focused on expanding access to testing through novel approaches, would ultimately bring together a diverse tactical team of experts representing every sector of government, academia, and industry. Collins stated in his testimony that *“RADx is engaging every scientist from the basement to the boardroom in an effort to improve current tests and advance completely new technologies.”* [9].

The reference to ‘Shark Tank’ in the title of the senate hearing is telling, as it implied a level of rigor and commercial diligence involved in deploying new technologies and a pace of development atypical in academic, peer-reviewed research. This ‘Shark Tank’ mentality would manifest as the ‘Deep Dive’ phase at the beginning of what would become the RADx Tech Innovation Funnel to Evaluate Testing Technologies for Covid-19 [10] (also see article by Dempsey *et al*. in this special issue). This funnel describes the stages of the process that BME academics and students participating on both sides of the process experience as either the proposal applicants and/or RADx Tech team members [10].

The RADx initiative resulted in unexpected learning opportunities for recent BME graduates, students, and faculty. Recent graduates were placed alongside industry veterans in an apprenticeship-like environment. Simultaneously, the rigor of the RADx Tech process on applicants submitting proposals provided an opportunity for academics to learn and appreciate the importance of professional skills in bringing solutions from BME research to commercial deployment [12].

This unexpected learning opportunity prompts the following questions about the impact of RADx Tech on learning and professional skill development. Can the RADx Tech structural model:

- Increase BME work readiness for current students and recent graduates who are proposal applicants and/or RADx Tech team members?
- Improve teaching of BME professional skills by faculty members who are proposal applicants?
- Serve as a model for BME professional skills development?

This paper opens with a literature review of the current status of BME professional skills development in traditional academic and clinical settings. We then provide an organizational structure of RADx Tech teams including the background and interactions within the process. Finally, we describe how RADx Tech can be leveraged for professional skills development and discuss potential opportunities for the future of such a model.

## II. Review of BME Professional Skills Development

Academic BME Programs were first introduced in the 1950s to bring together the engineering skills and understanding of biological systems needed to solve medical problems rooted in biology [13]-[15]. The diversity of academic and clinical practices brought together within the BME discipline, along with the importance of unmet needs in the medical space, provided a unique ecosystem for university-based innovation. In the United States, growth of BME programs across the country was initially fueled by leaders of foundations such as Whitaker and Wallace H. Coulter who together invested over $1BB in the creation of educational and research programs in BME [16]. This coincided with a period of growth in federal investment in engineering and medical research and the creation of the National Institute of Biomedical Imaging and Bioengineering (NIBIB) [17]. The degree also attracted significant additional investment in educational programming from governments, private entities, and institutions [18], [19]. In 2012, CNN Money called BME the best job in America, driving students to major in the discipline [20], [21].

The significant funding and interest in the BME major have predominantly been associated with the field’s promise for delivering medical innovations through meaningful, prosperous careers in both research and industry. One of the challenges of preparing graduates to enter industry roles is the predominance of traditional research-oriented faculty, often with limited industry exposure and experience navigating regulatory and commercial processes, and the need to train students for careers developing applied solutions in a regulated environment. The literature highlights the many different areas within the curriculum where these tensions emerge, and provides several possible contributing factors, including [4], [16], [19], [21]:

- Balancing the depth of technical skills versus the breadth of knowledge for such a diverse field, especially in a 4-year bachelor’s degree program [16], [22]
- Consistency among degree program offerings and the ranges of concentration areas such as imaging, informatics, biomechanics, biomaterials, tissue engineering, etc. [22], [23].
- Differing professional skills based on industry sectors such as medical devices, biologics, and pharmaceuticals [24].
- Academic programs ability to keep pace with a rapidly changing sector including the most pressing unmet needs, technology innovation, and regulatory landscape [15], [16], [22].

With such a complex multidisciplinary environment, the BME field has had a concerted effort over the past 20 years to fill these gaps and prepare the next generation of leaders. Below, we highlight four such efforts from the late 1990s and into the early 2000s as a case study of growth in the field of BME. This includes the Consortia for Improving Medicine with Innovation & Technology (CIMIT), the Stanford Biodesign Fellowship, the Coulter Translational Research Grant, and the establishment of the Biomedical Engineering Innovation Design and Entrepreneurship Alliance (BME-IDEA). Each of these programs identified collaboration across multiple disciplines, clinical immersion, and professional skills as key criteria for encouraging successful biomedical innovations [25]-[28].

CIMIT was formed in 1998 as a collaboration of academic research centers in the greater Boston area. Its founding was based on a recognition that facilitating early-stage collaboration between multidisciplinary and institutional teams was critical to achieve significant clinical impact [25]. Over the subsequent two decades, CIMIT matured from linking four research centers and expanded to a global network as a successful model for accelerating translational medical research through a collaborative effort.

That same year and 4000 miles to the west, the Medical Device Network, a precursor to Stanford Biodesign was launched. In particular, Stanford Biodesign highlighted the importance of bringing together multiple disciplines and clinical immersion. Their original program for postgraduates brought together an MD, BME PhD, MBA, and JD for a year-long immersive fellowship [29]. Since its founding, the Stanford model has been replicated and evolved with diverse variations implemented in the US and internationally taking shape in universities, hospitals, and inside corporate offices [30].

The Coulter Translational Research Grant focused on the collaboration of a BME faculty member with a practicing clinician to be eligible for the award. More importantly, the foundation provided professional skills training to the awardees of the grant known as Coulter College. Of particular note, the Coulter Process focused on a funnel approach to reduce technical, regulatory, market, intellectual property, and reimbursement risks [28]. Since its inception, the Coulter College evolved from a focus on grant awardees to training for undergraduate students and design faculty and further into the NIH Concept to Clinic: Commercializing Innovation (C3i) Program [31], [32].

BME-IDEA was formed to address a growing interest among BME faculty to share practices and learn together about how to apply these emerging approaches to design in their teaching. From an initial meeting of BME faculty interested in design and entrepreneurship organized by Stanford Biodesign and VentureWell in 2003, a community of faculty convened under VentureWell’s auspices regularly to disseminate curricular models, tools, and shared needs and interests. Among the resources that have emerged to address the needs identified by this group have been competitions for graduate and undergraduate students [28]. The NIBIB Design by Biomedical Undergraduate Teams (DEBUT) challenge is an annual competition organized in collaboration with VentureWell [33]. This competition recognizes undergraduate excellence in biomedical design and innovation and serves to highlight the collaboration and support by a private nonprofit and federal government to incentivize innovative academic programs. The BME-IDEA community has grown over the years spawning a network beyond the USA to encompass BME-IDEA networks in Europe, the Asia Pacific region, and most recently Africa. The topics of focus at network convenings has expanded substantially to include professional master’s degrees, doctorate programs, joint engineering and medicine programs, and fellowships [34].

In addition to these four programs, several other external organizations have contributed to the innovative growth seen within BME over the past 20 years. The National Science Foundation’s (NSF) Innovation Corps (I-Corps™) program has had an especially profound impact. This program, based on the LeanLaunch methodology developed by Steve Blank, provides student led teams an experiential learning opportunity focused on using customer discovery to identify and optimize the potential for a scientific or technological innovation to scale [35], [36]. The intensive team-based process has become ubiquitous in introducing students and faculty to entrepreneurship and the challenges of starting a business.

Today, BME professional skills training with a focus on innovation can be found in undergraduate, graduate, and postgraduate academic programs, at hospitals and clinics, in government-funded programs, and in professional organizations. They have emerged from the building blocks of CIMIT, Stanford Biodesign, Coulter Translational Research Grants, and BME-IDEA, and other foundational programs including I-Corps.

## III. Organizational Structure of RADx Tech Teams

**FIG. 1.**
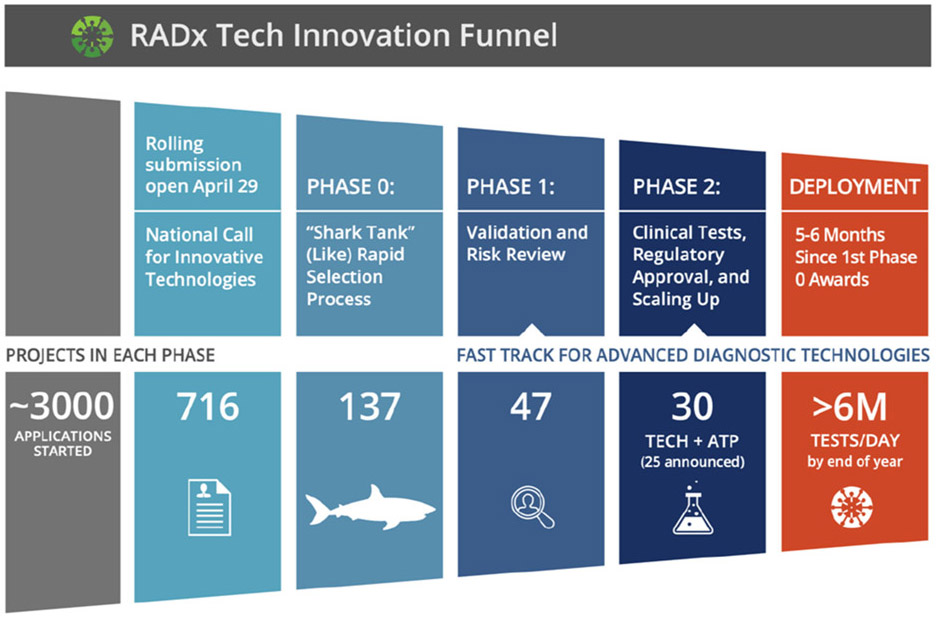
Rendition of the RADx Tech Innovation Funnel to Evaluate Testing Technologies for Covid-19 with data presented by Tromberg at the January 26th, 2021 RADx Tech monthly update [11].

The RADx Tech process brings together key elements from the foundation of biomedical professional skills. The process includes a funnel, as seen in the Coulter Commercialization Process [26]. Within each phase of the funnel is an iterative diligence cycle developed by CIMIT, the Guidance and Impact Tracking System (GAITS) [37]. GAITS ensures that key areas of risk reduction, such as clinical, market/business, regulatory, and technology, are progressing alongside product development. The innovation funnel developed by CIMIT for RADx Tech in conjunction with NIBIB opens with a weeklong intensive Phase 0 referred to as “shark tank” or “deep dive,” followed by a month-long Phase 1 focused on validation and risk reduction and closes with a Phase 2 focused on clinical testing, regulatory approval, and manufacturing scale up [10]. This funnel is previously shown in Fig. 1 and explained in detail by Dempsey *et al*. in this special issue.

**FIG. 2.**
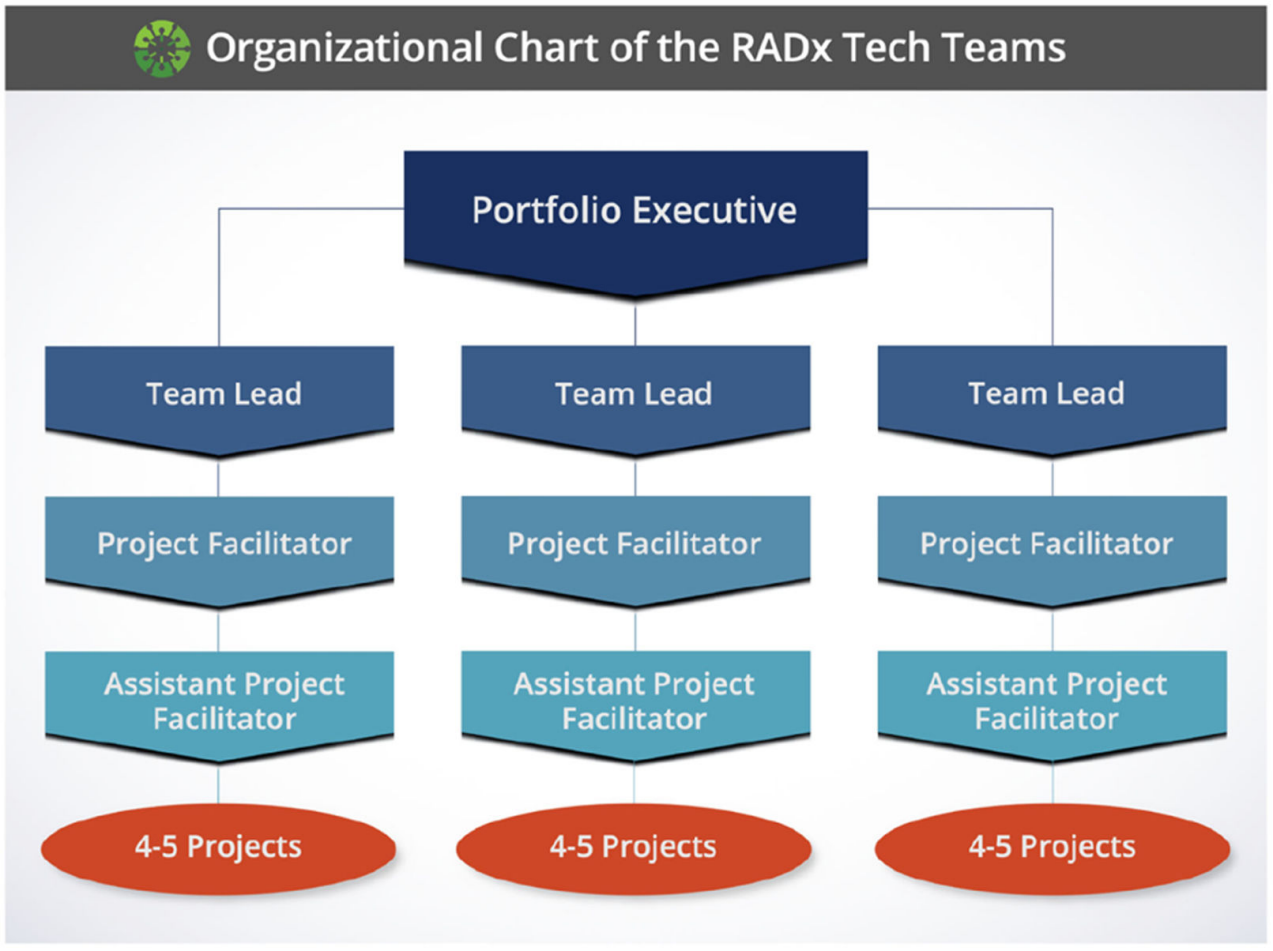
Organizational Structure of the RADx Tech Teams.

To implement this process, RADx Tech deploys teams of independent experts that perform both due diligence and support as applicants proceed through the innovation funnel. These teams are organized into a hierarchical structure of a Portfolio Executive (PE) that manages about four teams, each of which has three members including a Team Lead (TL), Project Facilitator (PF), and Assistant Project Facilitator (APF). The first teams were assembled in May of 2020. Through January 2021, the RADx Tech infrastructure included a total 28 teams managed by 6 PEs. Over this eight-month period, these teams implemented the funnel through 137 Deep Dives, 47 of which proceeded into Phase 1, and 30 of those into Phase 2 as shown in Fig. 1. For brevity and as shown in Fig. 2 , this organizational structure is referred to as ‘RADx Tech Teams.’ Throughout the first 5–6 months, each RADx Tech Team facilitated an average of 4 to 5 unique projects. The range of innovations required a breadth and depth of experiences and expertise, including seasoned entrepreneurs, scientists, and engineers from the In Vitro Diagnostics and Medical Devices sector. The efficiency of moving projects through the funnel demonstrated the organizational structure of RADx Tech was built to tackle the formidable challenge presented by the COVID-19 pandemic.

PEs serve as senior advisers and are drawn from a group of seasoned industry executives, serial entrepreneurs, and investors. TLs include a group of experts with typically 20 to 30 years of practical industry and entrepreneurial experience. While PFs often possessed similar depth and breadth of experiences as TLs, this role focused on technical expertise and project management skills. Rounding out this group is the APFs, of which the majority were recent graduates from BME programs. In the formative stages of building the RADx Tech Teams, it was recognized that including young professionals would be important to cultivate the next generation of leaders with experience in a pandemic response. It was also recognized that including these young professionals would be a value add due to their familiarity in managing a real-time multisite collaborative work environment.

## IV. Leveraging RADx Tech for Professional Skills Development

The potential educational impact of RADx Tech was a priority from the formation of the initiative in keeping with the educational mission of NIH. In May of 2020 with the support of the POCTRN centers, RADx launched a webinar series. These publicly available talks focused on topics from every aspect of the initiative, including regulatory pathways, technology innovation, clinical trials, and market considerations. Including the APFs in and of itself was a deliberate endeavor to train the next generation of biomedical leaders. That effort was quickly recognized as an invaluable experience for the APFs in professional skills development and work readiness. Most of the APFs with BME backgrounds had undergraduate senior design and professional master’s program experiences in managing the innovation process. For many of these early-career professionals, this experience served as a real world bootcamp from project proposal to commercial deployment.

It often takes several years in industry for early-career professionals to experience the entire lifecycle of biomedical innovation and to explore a broad variety of technologies and business models. In just a matter of months, APFs participated in an average of 4 to 5 proposals, representing technologies including polymerase chain reaction (PCR), lateral flow assay (LFA), enzyme-linked immunosorbent assay (Elisa), loop-mediated isothermal amplification (LAMP), agglutination, nucleic acid amplification test (NAAT), and other life science tools and collection devices. Business models included point-of-care diagnostics, reference labs, and over-the-counter testing. Each technology and business model were accompanied by a whole host of unique regulatory, clinical, and market challenges.

Academic translation programs that have an emphasis on professional skills development have a shared goal with the RADx Tech initiative in bringing innovations from the bench to the market. However, a differentiator for RADx Tech is an additional goal of seeing it through to that end. Developing strategies and nurturing innovations comprehensively to commercialization is important and often ambiguous work. Being part of an actual product development endeavor that gets to market is a unique experience that student and early-career APFs can learn from.

APFs were involved in a broad range of activities throughout different phases of the funnel and often managed three or more projects simultaneously. During the Phase 0 Deep Dive, APFs contributed to literature reviews, patent searches, competitive landscapes, and risk assessments that supported presentations by TLs to the RADx Tech steering panel. This type of work has previously been shown to contribute to work-readiness for early-career BME professionals [38]. In Phase 1, projects shifted to an accelerated technology verification and business risk reduction process. During this phase, teams vetted and developed plans, including technology performance, work plans for commercialization, quality management system requirements, product embodiment, and a use case analysis. The evaluation and planning processes for these criteria unfolded over a four- to six-week period and included detailed activities for each criterion. For example, the work plan for commercialization included aspects such as scaleup plans for production volumes, timelines, milestones, supply chain management, and budgeting. Notably, the use case analysis included an assessment by a clinical review board consisting of seasoned medical practitioners and scientists. APFs played a key role in information management throughout this rapid phase of the RADx Tech process and experienced first-hand feedback from such expert reviews. As projects moved into the scaleup and commercialization activities of Phase 2, the RADx Tech teams focus shifted from due diligence and planning to a role of project management. APFs often had the primary responsibility of producing and managing quad-charts, tri-charts, and databases to communicate accomplishments, challenges, and support to ensure projects were keeping up with aggressive timelines and meeting their milestones.

Throughout all phases of the RADx Tech funnel, APFs participated in leadership meetings, viability review feedback sessions, and a host of training sessions for managing a wide array of information and reporting infrastructure such as GAITS. In addition to learnings from the PEs, TLs, and PFs on their own project teams, APFs also attended meetings that included external stakeholders and consultants. These included C-suite and director level executives, FDA representatives, regulatory consultants, and quality systems experts. Throughout the process, the APFs supported companies to establish good manufacturing practices (GMP) and prepare for related certifications such as ISO 9001 and 13485. They supported teams in developing clinical studies for emergency use authorization (EUA) submissions to the FDA. They helped produce quality systems documents and procedures such as hazard analysis and design history requirements. The educational value of the APF experience throughout the RADx Tech initiative is presumed substantial and further investigation is needed to better understand the full significance.

**FIG. 4.**
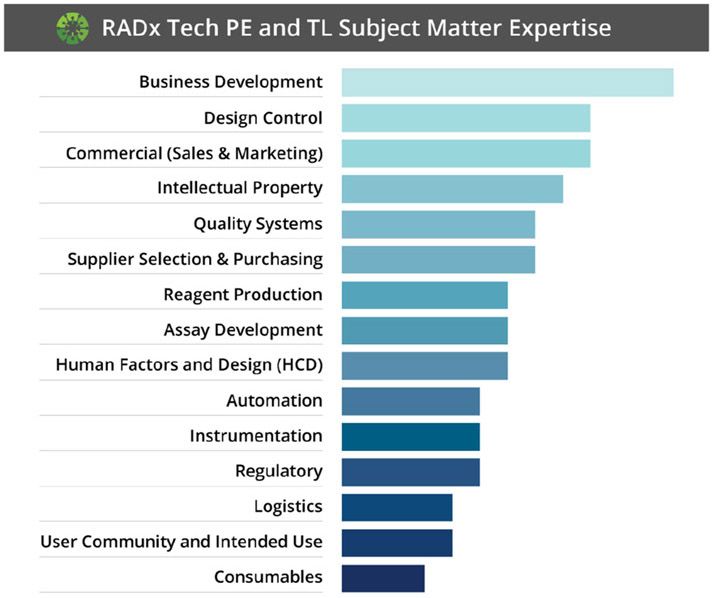
Visual showing the combined professional skills of RADx TLs and PEs (N=53). The bar chart represents a weighted average of expertise on a scale of 1 to 4, where 1 is basic knowledge and 4 is subject matter expert.

Further, the RADx Tech initiative provided learning opportunities for more than just the APFs. Deep learning was also experienced by PEs, TLs, and PFs throughout the RADx Tech initiative. Furthermore, this learning environment was not only seen within the RADx Tech teams, but also among team-to-team interactions. In the first month of RADx Tech, the breadth and depth of experience was recognized and a contact list of PEs and TLs was assembled noting expertise across the group. If one team was missing a key element, there was rarely a time that they needed to reach outside of this network to find a subject matter expert. The breadth and depth of this expertise is shown in Fig. 4.

The educational culture reached beyond public webinars and RADx Tech teams. Significant professional skills development was afforded to the applicants in the innovation funnel. Many of the applicants themselves were from the ranks of academia including faculty and graduate students. The Phase 0 Deep Dive process emphasized performing diligence and making go-forward decisions not by technology alone, but considering all aspects of market potential, regulatory burden, and clinical testing needed to deploy. For many applicants with strong track records of funding, it was often the barriers related to professional skills, not technology, that proved to be the biggest challenges for proceeding or not advancing through the innovation funnel.

## V. Opportunities Ahead

The papers included in this special issue highlight many benefits and opportunities of the RADx Tech initiative. There is significant opportunity for lessons learned during the COVID-19 pandemic to improve professional skills development for the future generation of biomedical innovators and leaders. Current faculty members that are exposed to the RADx Innovation Funnel may begin to fully appreciate the importance of training for development of applied solutions in a regulated environment and minimize the existing tension with a traditional research orientation. This could be achieved if new funding mechanisms that have a focus on translation utilized the RADx Tech structural model to vet, make funding decisions, and support meritorious projects.

In such an environment, proposals not selected for awards would have the benefit of feedback afforded by the process. Those that are awarded would then have the chance for collaboration with the experienced members of the RADx Tech Teams. On those teams, the BME graduates serving as APFs will experience the immersive professional skills training as described in this paper. The NIH has a history of supporting professional skills development in BME, as exemplified by their collaborations in the DEBUT competition, the C3i program, and with I-Corps™. One such opportunity for a sustainable model in furthering the education of the best and brightest BME graduates is to develop an internship program for DEBUT winners that can then serve APFs in a future rendition of the RADx Tech structural model.

## VI. Conclusion

There are many benefits of the RADx Tech initiative well worth exploring beyond that of the current acceleration of diagnostic tests being developed and deployed to the nation. One of those benefits has been the impact on work readiness for recent BME graduates who have been hired as APFs and to the students and faculty members on applicant teams. This model builds on the exposure to design and innovation that is now a standard part of the BME curriculum by providing an intensive immersion in biomedical product development.

Imagine a world where COVID-19 is behind us and RADx Tech lives on. Is the RADx Tech organizational structure, funnel process, and integration of APFs a replicable value add to improve health and advance the nation’s economic wellbeing? To that end, a survey study is planned. Opportunities such as a RADx Tech APF post-graduate fellowship may offer a sustainable model to enhance work readiness for the next generation of biomedical leaders. The RADx Tech innovation funnel process can provide an educational opportunity for the faculty and students participating in such a program if sustainable beyond COVID-19.

## Figures and Tables

**FIG. 3.**
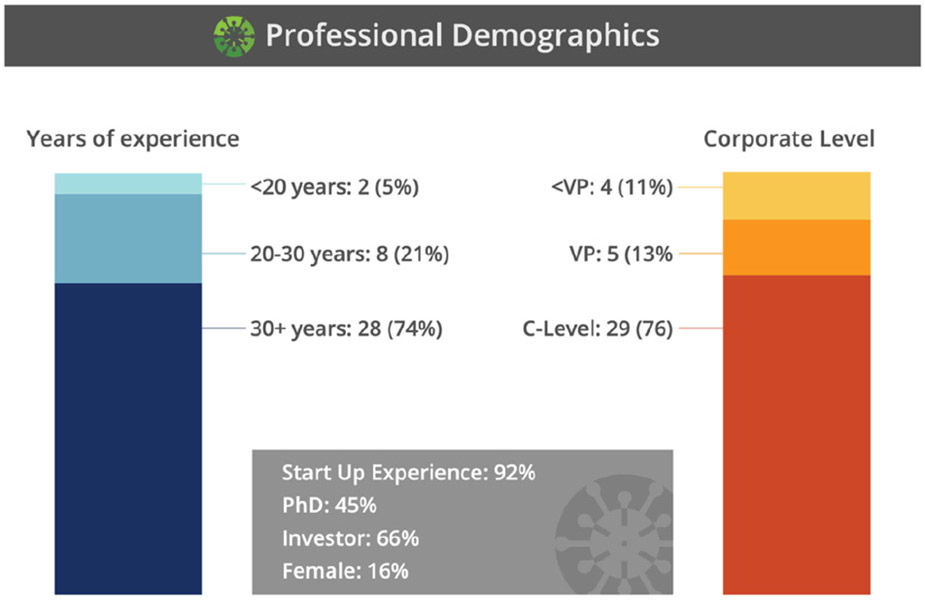
The experience of the PEs and TLs as presented by Dempsey at the January 26th, 2021 RADx Tech monthly update [11].

## Impact Statement

An unanticipated outcome of the COVID-19 pandemic and RADx Tech response was a unique experience addressing gaps in professional skills education that has been a perennial challenge for BME programs.

## References

[1] Baura G, “Educating for industry: A call to action for bio-/biomedical engineering professors and students,” IEEE Pulse, vol. 6, no. 2, pp. 5–9, 4. 2015, doi: 10.1109/mpul.2014.2386471. 25946757

[2] Kirsch RF, LaBerge M, Perreault EJ, and King MR, “Announcing the fourth biomedical engineering education summit meeting,” Cel. Mol. Bioeng, vol. 12, no. 2, pp. 135–138, 4. 2019, doi: 10.1007/s12195-019-00568-1. PMC6816753 31719905

[3] Baura G, and Tiffany Berry PD, “Comprehensive teaching of medical devices,” Vancouver, BC, 6. 2011, pp. 22.359.1–22.359.10, doi: 10.18260/1-2-17640.

[4] Rivera CP, Huang-Saad A, Woodcock CSE, and Wang A, “Preparing early-career biomedical undergraduates through investigations of stakeholder needs: A qualitative analysis,” presented at ASEE Virtual Annu. Conf., Online, 6. 2020, doi: 10.18260/1-2-35079.

[5] Huang-Saad A, and Springer E, “Transforming biomedical engineering education through instructional design,” IJEE Int. J. Eng. Educ, vol. 36, no. 3, 1. 2020, Accessed: Feb. 08, 2021. [Online]. Available: https://par.nsf.gov/biblio/10159108-transforming-biomedical-engineering-education-through-instructional-design

[6] White JA , “Core competencies for undergraduates in bioengineering and biomedical engineering: Findings, consequences, and recommendations,” Ann Biomed Eng, vol. 48, no. 3, pp. 905–912, 3. 2020, doi: 10.1007/s10439-020-02468-2. 32026231 PMC13082399

[7] Zamora-Ledezma C , “Biomedical science to tackle the COVID-19 pandemic: Current status and future perspectives,” Molecules, vol. 25, no. 20, 10. 2020, doi: 10.3390/molecules25204620. PMC7587204 33050601

[8] Armani AM, Hurt DE, Hwang D, McCarthy MC, and Scholtz A, “Low-tech solutions for the COVID-19 supply chain crisis,” Nature Rev. Mater, vol. 5, no. 6, 6. 2020, Art. no. 6, doi: 10.1038/s41578-020-0205-1. PMC7212509 32395258

[9] Collins F, Tank S, “New tests for COVID-19 ∣ the U.S. Senate Committee on Health, Education, Labor and Pensions,” 106 Dirksen Senate Office Building, Washington, DC, 2020.

[10] Tromberg BJ , “Rapid scaling up of covid-19 diagnostic testing in the united states — The NIH RADx initiative,” New England J. Med, vol. 383, no. 11, pp. 1071–1077, 9. 2020, doi: 10.1056/NEJMsr2022263. 32706958 PMC7493127

[11] Tromberg B, Dempsey M, Schachter S, and Heemskerk J, “RADx tech monthly update,” 1. 26, 2021, [Online]. Available: https://www.poctrn.org/-/meeting-radx-tech-combined-working-group

[12] Berglund J, “The real world: BME graduates reflect on whether universities are providing adequate preparation for a career in industry,” IEEE Pulse, vol. 6, no. 2, pp. 46–49, 3. 2015, doi: 10.1109/MPUL.2014.2386631. 25782113

[13] Linsenmeier RA, “What makes a biomedical engineer?,” IEEE Eng. Med. Biol. Mag, vol. 22, no. 4, pp. 32–38, 7. 2003, doi: 10.1109/MEMB.2003.1237489. 14515690

[14] Harris TR, Bransford JD, and Brophy SP, “Roles for learning sciences and learning technologies in biomedical engineering education: A review of recent advances,” Annu. Rev. Biomed. Eng, vol. 4, no. 1, pp. 29–48, 2002, doi: 10.1146/annurev.bioeng.4.091701.125502. 12117749

[15] Huang-Saad A, Stegemann J, and Shea L, “Developing a model for integrating professional practice and evidence-based teaching practices into BME curriculum,” Ann. Biomed. Eng, vol. 48, no. 2, pp. 881–892, 2. 2020, doi: 10.1007/s10439-019-02427-6. 31811475

[16] Katona PG, “Biomedical engineering and the whitaker foundation: A thirty-year partnership,” Ann. Biomed. Eng, vol. 34, no. 6, pp. 904–916, 6. 2006, doi: 10.1007/s10439-006-9087-7. 16676132

[17] Hendee WR, Chien S, Maynard CD, and Dean DJ, “The national institute of biomedical imaging and bioengineering: History, status, and potential impact,” Ann. Biomed. Eng, vol. 30, no. 1, pp. 2–10, 1. 2002, doi: 10.1114/1.1433491. 11874139

[18] DiMeo AJ, and DesJardins JD, “Editorial notes,” Ann. Biomed. Eng, vol. 41, no. 9, pp. 1801–1802, 9. 2013, doi: 10.1007/s10439-013-0884-5. 23925471

[19] Pecchia L, Pallikarakis N, Magjarevic R, and Iadanza E, “Health technology assessment and biomedical engineering: Global trends, gaps and opportunities,” Med. Eng. Phys, vol. 72, pp. 19–26, 10. 2019, doi: 10.1016/j.medengphy.2019.08.008. 31554572

[20] Ashford K, “Best jobs in america: CNN Money/PayScale.com’s list of great careers,” CNNMoney, 10. 29, 2012, Accessed: Feb. 08, 2021. [Online]. Available: https://money.cnn.com/pf/best-jobs/2012/snapshots/index.html

[21] National Academy of Engineering, “Major findings and recommendations,” in Understanding the Educational and Career Pathways of Engineers. Washington, DC, USA: The National Academies Press, 2018, pp. 117–124.

[22] Enderle JD, and Bronzino JD, Introduction to Biomedical Engineering. Third. Burlington, MA, USA: Academic, 2012.

[23] Linsenmeier R, and Gatchell D, “Core elements of an undergraduate biomedical engineering curriculum–State of the art and recommendations,” presented at the 9th Int. Conf. Eng. Education, San Juan, Puerto Rico, 7. 2006, pp. 22–24.

[24] Rohde J, France J, Benedict B, and Godwin A, “Exploring the early career pathways of degree holders from biomedical, environmental, and interdisciplinary/multidisciplinary engineering,” presented at the ASEE Virtual Annu. Conf., 6. 2020, doi: 10.18260/1-2-34646.

[25] Schachter SC, Collins J, Dempsey MK, Spiliotis D, and Parrish J, “Deep innovation in the medical domain a la boston’s CIMIT: Institutional case study,” Venture Findings, vol. 3, pp. 21–30, 2016.

[26] Brinton TJ , “Outcomes from a postgraduate biomedical technology innovation training program: The first 12 years of stanford biodesign,” Ann. Biomed. Eng, vol. 41, no. 9, pp. 1803–1810, 9. 2013, doi: 10.1007/s10439-013-0761-2. 23404074 PMC3759560

[27] Marten T, “Funding engineering/surgical partnerships to accelerate commercialization of academic medical and surgical innovations: The coulter model for translational partnership between medicine, engineering, and industry,” in Success in Academic Surgery: Innovation and Entrepreneurship, Cohen MS and Kao L, eds., Cham, Switzerland: Springer International Publishing, 2019, pp. 159–184.

[28] Goldberg J, “Preparing students for medical device innovation: Notes from BME-IDEA 2018,” IEEE Pulse, vol. 10, no. 1, pp. 32–35, 1. 2019, doi: 10.1109/MPULS.2018.2885834.

[29] Leslie M, “Medical device network aids inventors: 12/98,” Stanford Report, 12. 09, 1998, Accessed: Feb. 08, 2021. [Online]. Available: https://news.stanford.edu/news/1998/december9/mdn129.html

[30] Wall J , “The impact of postgraduate health technology innovation training: Outcomes of the stanford biodesign fellowship,” Ann. Biomed. Eng, vol. 45, no. 5, pp. 1163–1171, 5 2017, doi: 10.1007/s10439-016-1777-1. 28004213 PMC5397448

[31] Merchak T, and Goldberg I, “Concept to clinic: Commercializing innovation (C3i) program,” 2021, Accessed: Feb. 08, 2021, [Online]. Available: https://www.nibib.nih.gov/research-program/c3i-program

[32] MacDougall RA, “The art of sealing the biomedical technology deal,” Nat. Inst. Biomed. Imag. Bioeng. (NIBIB), 6. 21, 2017, Accessed: Feb. 08, 2021, [Online]. Available: https://www.nibib.nih.gov/news-events/newsroom/art-sealing-biomedical-technology-deal

[33] Erim Z, “Design by biomedical undergraduate teams (DEBUT) challenge,” Nat. Inst. Biomed. Imag. Bioeng, 2021, Accessed: Feb. 08, 2021, [Online]. Available: https://www.nibib.nih.gov/research-programs/DEBUT-challenge

[34] Engel JS, Global Clusters of Innovation: Entrepreneurial Engines of Economic Growth Around the World. Cheltenham, U.K.: Edward Elgar Pub, 2016.

[35] Colao JJ, “Steve blank introduces scientists to a new variable: Customers,” Forbes, 8. 01, 2012, Accessed: Feb. 08, 2021, [Online]. Available: https://www.forbes.com/sites/jjcolao/2012/08/01/steve-blank-introduces-scientists-to-a-new-variable-customers/

[36] Nnakwe CC, Cooch N, and Huang-Saad A, “Investing in academic technology innovation and entrepreneurship: Moving beyond research funding through the NSF I-CORPSTM program,” Technol. Innov, vol. 19, no. 4, pp. 773–786, 6. 2018, doi: 10.21300/19.4.2018.773.

[37] Collins J, “Guidance and impact tracking system (GAITS),” 2021, Accessed: Feb. 08, 2021, [Online]. Available: https://www.gaits.org/

[38] Carroll AJ, Hallman SJ, Umstead KA, McCall J, and DiMeo AJ, “Using information literacy to teach medical entrepreneurship and health care economics,” J. Med. Library Assoc, vol. 107, no. 2, pp. 163–171, 4. 2019, doi: 10.5195/jmla.2019.577. PMC6466497 31019384

